# Reducing central vein catheterization complications with a focused educational program: a retrospective cohort study

**DOI:** 10.1038/s41598-020-74395-0

**Published:** 2020-10-16

**Authors:** Laryssa P. T. Hanauer, Pedro H. Comerlato, Afonso Papke, Marina Butzke, Andressa Daga, Mariana C. Hoffmeister, Marcio M. Boniatti, Josiane F. John, Beatriz D. Schaan, Dimitris V. Rados

**Affiliations:** 1 Internal Medicine Service, Hospital de Clinicas de Porto Alegre (HCPA), Rua Ramiro Barcelos 2350, Sala 700, Porto Alegre, Rio Grande do Sul CEP 90035-903 Brazil; 2 Intensive Care Service, HCPA, Porto Alegre, RS Brazil; 3 Universidade Federal do Rio Grande do Sul (UFRGS) School of Medicine, Porto Alegre, RS Brazil; 4 Endocrinology Service, HCPA, Porto Alegre, RS Brazil

**Keywords:** Health care, Medical research, Epidemiology

## Abstract

Central venous catheters (CVCs) are frequently used, but the rate of complications is high. This study evaluates the effects of a short training program for CVC insertion in a university-based teaching hospital. A sample of adults with CVCs inserted outside the intensive care unit was selected from two academic years: 2015, year without structured training, and 2016, year with structured training. Clinical and laboratory information, as well as the procedure’s characteristics and complications (mechanical and infectious) were collected. The incidence of complications before and after the training was compared. A total of 1502 punctures were evaluated. Comparing the pre- and post-training period, there was an increase in the choice for jugular veins and the use of ultrasound. A numerical reduction in the rate of complications was identified (RR 0.732; 95% CI 0.48–1.12; *P* = 0.166). This difference was driven by a statistically significant lower rate of catheter-related infections (RR 0.78; 95% CI 0.64–0.95; *P* = 0.047). In the multivariate analysis, aspects regarding technique (ultrasound use, multiple punctures) and year of training were associated with outcomes. Structured training reduces the rate of complications related to CVC insertion, especially regarding infections.

## Introduction

Short-term central venous catheters (CVCs) are of vital importance for the diagnosis and treatment of hospitalized patients with the most diverse clinical conditions^1^. However, the rate of complications associated with the insertion procedure is high. It is a significant cause of preventable morbidity and mortality^2^. They include catheter infection, pneumothorax, hemothorax, and guidewire loss^2–4^. The use of the subclavian vein is more related to pneumothorax and less related to infections, and the internal jugular vein and femoral vein are more related to arterial perforation^3,5^. A previous evaluation at our hospital showed that mechanical complications occurred in 6.5% of procedures and infection complications occurred in 11.1% of procedures^6^.

Preventive measures to avoid these complications are being increasingly studied and recommended. The use of antisepsis and infection prevention bundles has decreased the incidence of infections related to these procedures^7,8^. Besides, to prevent mechanical complications, an increasing number of insertions have been conducted under ultrasonography, leading to a lower rate of complications^8^. Unfortunately, this resource is still not widely available in Brazil or other low- or middle-income countries. Also, lack of training limits is use, even when the device is available. This resource was proven effective only when more experienced professionals conducted the procedure^9,10^. Other interventions have been tested, such as educational programs. For example, an intervention comprising multi-modal structured training program integrated with a modified, pre-packed CVC set and drapes with reminder stickers eliminated guidewire retention during CVC insertion^11^.

Considering the need for continuous medical education and its potential for reducing medical complications of CVC insertion, especially for physicians in training, the hospital’s management decided to put into action an education program in this procedure. The objective of this study was to compare the rate of complications before (the academic year of 2015) and after (the academic year of 2016) this education program.

## Methods

This study is a retrospective cohort of adult patients conducted at a tertiary care teaching hospital in Southern Brazil, a middle-income area. It is an 842-bed hospital and state reference center for the treatment of many high-complexity health-related conditions where several medical residency programs take place. All methods were carried out in accordance with relevant guidelines and regulations of Hospital de Clínicas de Porto Alegre research ethics committee, which also approved the study. Hospital de Clínicas de Porto Alegre research ethics committee waived the need of the written informed consent. We followed recommended protocols to deal with patients’ medical charts and all researchers involved in data handling provided written commitment to data safety.

Electronic health records (EHR) were retrospectively reviewed. Through the computerized management system, we searched adult patients who received a CVC during the 2015 and 2016 academic years outside the intensive care unit (ICU). The academic year comprises March of one year until February of the subsequent year. We used radiographs to identify the patients, as they are routinely performed in all patients after jugular or subclavian catheterization. From this initial search, a random sample was selected for evaluation using a random number list. Exclusion criteria were radiography that did not follow a new procedure (for example, after accidental traction or catheter exchange by guidewire), radiography performed on inpatients admitted to the ICU, peripherally inserted CVCs, and CVCs placed via the femoral vein.

The provision of structured training on CVC insertion was a decision of the hospital’s management, aligned with institutional actions to improve patient safety in the last few years. The training was outlined based on current evidence regarding the insertion of central venous access devices^12^, and provided for the new residents (first-year residents). It was divided into three steps during the first month of residence: a 60-min lecture, practical training in mannequins via anatomical landmarks (location of jugular, subclavian, and femoral veins) plus ultrasound handling, and a 10-question theoretical test. In addition to the technique, concepts of bundles for infection prevention and care with central lines were approached. The local protocol includes qualified personnel involved in catheter changing and care, good hand hygiene, use of an alcoholic formulation of chlorhexidine for skin disinfection, and manipulation of the vascular line. Also, the training stimulates avoiding the femoral vein route for the insertion of CVCs; preference for ultrasound guidance rather than landmark method; using full-barrier precautions during insertion; removal of unnecessary catheters.

Ultrasound guidance is strongly recommended. However, it cannot be mandatory in our setting (middle-income country), as not every hospital provides this device, and residents must be trained to deal with different situations. These new residents were only given authorization to insert CVCs after they completed all these steps, and a preceptor or senior resident supervised their first procedures.

Data were collected from EHR using an electronic template. The following information was collected: gender, age, platelet count, prothrombin time, cardiac or pulmonary disease, infection, neoplasm, renal, neurological or liver disease, and diabetes mellitus. These comorbidities were recorded if reported at hospital discharge or patient death notes. The following data related to the CVC and procedure were collected: successful or unsuccessful catheterization, type of CVC (monolumen, doublelumen, hemodialysis, indwelling), CVC indication, area of the physician who carried out the procedure (medical or surgical), resident length of training (grouped by less than two years or more than two years in residency), where the procedure took place (ambulatory surgical center, main surgical center or other), shift (day: 8 am–8 pm; or night: 8 pm–8 am), use of ultrasound, insertion site (jugular or subclavian), and multiple attempts. The periods evaluated were March 2015 to February 2016 (2015, year with no structured training) and March 2016 to February 2017 (2016, year with structured training).

The procedure-related complications were categorized as mechanical (arterial perforation, hematoma, pneumothorax, thrombosis) or infectious (catheter-related infection). The data related to mechanical complications were collected in the description of the procedure, in the postprocedural radiological report, and subsequent medical or nursing staff entries (up to 7 days). The catheter-related infection data were obtained from the hospital infection control committee, observing the criteria of the Brazilian National Health Surveillance Agency^13^. They are defined as an adult patient bearing central venous catheter at diagnosis or within 48 h after its removal who presented (1) one or more positive blood cultures for a recognized pathogen unrelated to infection elsewhere or (2) temperature, chills, oliguria, or hypotension concurrent with at least two blood cultures harvested at various times and positive for a skin-contaminating pathogen unrelated to infection elsewhere. The incidence of infectious complications included an event occurring between the insertion of the catheter and hospital discharge or death.

### Statistical analysis

Continuous variables are presented as mean and standard deviation, and categorical variables are reported in percentage and absolute number. Comparisons of years with and without structured training were conducted using the chi-square test. Multivariate analysis was conducted to control for literature-established confounding factors and those found in our previous study (multiple attempts, first or second semester), and differences in baseline characteristics between the years (insertion site and ultrasound use; in 2016 increased use of jugular vein and ultrasound). These analyses were conducted using SPSS 20.0 software (SPSS, Chicago, IL). Considering that in the previous study, the rate of complications of CVC insertion in the same hospital was 6%^6^, a sample size of 749 procedures was estimated for each year to identify an absolute 3% reduction in the risk of complications related to CVCs.

## Results

**Figure 1:**
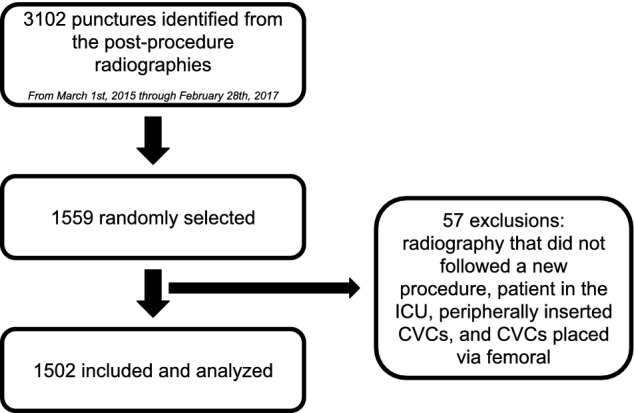
Flowchart of selected cases.

A total of 3102 postprocedure radiographs after CVC insertion was identified in adult patients outside the ICU from 2015 to 2016. Of these, 1559 cases were randomly selected for evaluation, of which 57 were excluded (Fig. 1). For the current analysis, 1502 procedures were included, 754 in year 1 (2015) and 748 in year 2 (2016).

The characteristics of the patients studied are depicted in Table 1. The samples were homogeneous and included similar number of men and women with a mean age of 53 years. The main indications for CVC insertion were inaccessible peripheral veins and the need for chemotherapy. Most of the procedures were carried out by physicians with less than 2 years of medical training (first- and second-year residents). Neoplastic disease was the most identified morbidity. From 2015 to 2016, the use of ultrasound and the jugular vein increased, and elective procedures decreased (portocath).

**Table 1:** Characteristics of patients included and procedures—comparison of 2015 (no training provided) and 2016 (training provided).

	2015 (n = 754)	2016 (n = 748)
Age (years)	53 ± 17	55 ± 16
Female gender	375 (49.9%)	409 (54.8%)
INR > 1.5	33 (4.4%)	44 (5.9%)
Platelet count < 50,000	43 (5.7%)	28 (3.7%)
Infection	71 (9.6%)	111 (15.5%)
Heart disease	78 (10.6%)	115 (16%)
Lung disease	67 (9.1%)	60 (8.3%)
Neoplasm	439 (59.5%)	327 (45.5%)
Renal disease	149 (20.2%)	238 (33.1%)
Diabetes mellitus treated with insulin	64 (8.7%)	78 (10.8%)
Neurologic disease with functional limitation	108 (14.6%)	95 (13.2%)
Liver disease	35 (4.7%)	41 (5.7%)
**Catheter indication**		
No peripheral veins	225 (35%)	212 (31.3%)
Sepsis/shock	75 (11.7%)	77 (11.4%)
Large surgery	55 (8.6%)	120 (11.7%)
Dialysis	70 (10.9%)	114 (16.8%)
Chemotherapy	206 (32.1%)	139 (20.5%)
Others	11 (1.7%)	16 (2.4%)
**Area of the professional responsible for the procedure**		
Clinical	129 (17.5%)	164 (22.7%)
Surgical	564 (76.4%)	515 (71.4%)
**Resident length of training**		
Less than 2 years	533 (77.7%)	504 (77.4%)
More than 2 years	153 (22.3%)	147 (22.6%)
**Situation**		
Ambulatory	93 (12.6%)	48 (6.6%)
In hospital	645 (87.4%)	675 (93.4%)
**Location of the procedure in the hospital**		
Surgical center	587 (80.1%)	550 (76%)
Ward or Emergency Department	146 (19.9%)	174 (24%)
**Ultrasound use**		
Yes	255 (33.8%)	299 (40%)
No or not described	499 (66.2%)	449 (60%)
**Insertion site**		
Jugular	429 (58.4%)	536 (74.2%)
Subclavian vein	305 (41.6%)	186 (25.8%)

Data reported in mean ± standard deviation or N (%). N (2015) ranged from 642 to 754. N (2016) ranged from 678 to 748.

The incidence of complications, mechanical or infectious, in 2015 compared to 2016 is depicted in Table 2. The rate of complications decreased from 7.2 to 5.3% when the years with and without training were compared; however, this decrease was not statistically significant (OR 0.732; 95% CI 0.48–1.12; *P* = 0.166). The incidence of mechanical complications was 2.7% in both years; this pattern was sustained when we specified the outcome (Table 2). Fewer complications arose due to infection in the 2016 academic year. In absolute terms, the reduction was 3%, representing an odds ratio of 0.784 (95% CI 0.642–0.957; *P* = 0.047).

**Table 2:** Risk of complications according to the training year (2015, no training provided; 2016, training provided).

	Academic year 2015	Academic year 2016	Odds ratio	CI (95%)	P
Any complication	54 (7.2%)	40 (5.3%)	0.732	0.480–1.117	0.16
Mechanical complication	20 (2.7%)	20 (2.7%)	0.996	0.727–1.363	1.000
Arterial injury	13 (2.4%)	11 (1.8%)	0.734	0.326–1.651	0.537
Hematoma	6 (1.1%)	7 (1.1%)	1.043	0.348–3.122	1.000
Pneumothorax	3 (0.4%)	6 (0.8%)	2.062	0.514–8.275	0.336
Infection	38 (5%)	22 (2%)	0.784	0.642–0.957	0.047

“Any complication” includes the sum of all mechanical and infectious complications.

Data reported in N (%).N (2015) ranged from 563 to 754. N (2016) ranged from 563 to 748.

The multivariate analysis for the predefined outcomes is presented in Table 3. After we controlled for confounders (academic year, use of ultrasound, insertion site, multiple attempts, and semester), training had no association with the mechanical and infectious complications. On the other hand, when the analysis specified mechanical or infectious complications, structured training was associated with fewer infections. Moreover, a single puncture was a protective factor for mechanical complications (OR 0.037; 95% CI 0.018–0.077; *P* < 0.001), and not using ultrasound was associated with an increased chance of any complications (OR 1.704; 95% CI 1.028–2.825; *P* = 0.03).

**Table 3:** Multivariate analysis of complications.

Independent variables	Odds ratio	CI (95%)	*P*
**Any complication**			
Academic year 2015	1.449	0.937–2.441	0.95
No ultrasound	1.704	1.028–2.825	0.03
Jugular vein	1.052	0.677–1.633	0.09
Single attempt	0.172	0.105–0.280	< 0.001
First semester	1.052	0.677–1.633	0.82
**Mechanical complication**			
Academic year 2015	1.057	0.540–2.070	0.87
No ultrasound	1.446	0.664–3.152	0.35
Jugular vein	1.452	0.608–3.468	0.40
Single attempt	0.037	0.018–0.077	< 0.001
First semester	1.597	0.774–3.295	0.20
**Infectious complication**			
Academic year 2015	1.797	1.045–3.092	0.03
No ultrasound	1.631	0.884–3.009	0.11
Jugular vein	1.651	0.886–3.073	0.11
Single attempt	0.782	0.344–1.777	0.55
First semester	0.795	0.472–1.340	0.38

“Any complication” includes the sum of all mechanical and infectious complications; academic year 2015 compared to academic year 2016; no ultrasound compared to ultrasound use; jugular vein compared to subclavian vein; single attempt compared to more than one attempt; first semester of academic year compared to second semester of academic year.

## Discussion

The present study showed that the focused education program provided for first-year residents for CVC insertion in a tertiary hospital significantly reduced the rate of catheter-related infections, and no difference emerged in the overall incidence of mechanical complications. The use of ultrasound and the number of attempts were determining factors for the presence of any complications. These results support the importance of training residents in the insertion of CVCs^12,14^.

CVC-related bloodstream infections are the fourth leading cause of hospital-acquired infection and occur in about 4–35% of procedures^15,16^. This study found a 22% relative reduction in the rate of catheter-related infections 1 year after an educational program was implemented in a tertiary hospital. This finding is in accordance with data from the literature, which shows that systematized training of medical interns and residents can reduce CVC-related infectious complications by up to 84%^14^. The fact that this result is consistent when we insert other variables into the model reinforces the result. We believe that this difference may be related to improved knowledge of and compliance with the bloodstream infection bundle included in the proposed training program^17^. Despite the positive results related to the bundle, a study our hospital conducted found that this bundle is completely carried out in only 54.4% of the procedures conducted in ICUs^18^. The current training has likely increased adherence to these measures. Furthermore, the adherence to infection prevention bundles is probably a cost-effective intervention: it requires only the better use of the available resources, but no additional investments besides training.

The most frequent mechanical complications are arterial perforation, hematoma, and pneumothorax^3,4^. Pneumothorax, a complication with high morbidity, is rare, occurring in less than 1% of the jugular procedures and no more than 1.5% of the subclavian ones^5^. Our cohort has a greater rate of mechanical complications, but this may be related to a different context. Ultrasound use is just scaling up and this seems to lead to significant reductions in mechanical complications^19^. Also, we are dealing with in-training physicians, rather than experienced practitioners^5^.

In our results, we stated that multiple puncture attempts increase the risk of mechanical complications. The training proposed in 2016 did not modify this type of complication. We understand that this finding reinforces the importance of optimal surgical technique to prevent mechanical complications. The multivariable analysis of our results demonstrates that using it was related to the reduction of all complications. Currently, the use of ultrasound for invasive procedures is encouraged throughout the hospital to reduce complications, as the literature has demonstrated^20–22^. Adequate and directed training on the use of the device is crucial for the procedure’s success. Another benefit of ultrasound use may be the confirmation of the catheter position post-procedure, although the sensitivity may be as low as 55%^23^.

The study has limitations, among them the retrospective design that may hinder the recovery of outcomes and related factors, due to underreporting in the medical record. They may be restricted to similar hospitals and healthcare systems in low- and middle-income countries. Another limitation may be that other care initiatives were implemented in the hospital during the evaluation period and may have also impacted the results.

## Conclusion

This study reinforces the steps to reduce incidents related to central venous puncture. Theoretical-practical training with several didactic strategies (theoretical class, training in mannequins and ultrasound, subjective content test, asepsis protocols, and surgical technique) leads to better procedural outcomes, mainly reducing infectious complications.
